# Effect of sweet potato purple acid phosphatase on *Pseudomonas aeruginosa* flagellin-mediated inflammatory response in A549 cells

**DOI:** 10.5713/ab.22.0059

**Published:** 2022-06-30

**Authors:** Heyeon Baik, Jaiesoon Cho

**Affiliations:** 1 Department of Animal Science and Technology, Konkuk University, Seoul 05029, Korea

**Keywords:** A549 Cell, Flagellin, Inflammatory Response, *Pseudomonas aeruginosa*, Sweet Potato Purple Acid Phosphatase

## Abstract

**Objective:**

The study was conducted to investigate the dephosphorylation of *Pseudomonas aeruginosa* flagellin (PA FLA) by sweet potato purple acid phosphatase (PAP) and the effect of the enzyme on the flagellin-mediated inflammatory response in the A549 lung epithelial cell line.

**Methods:**

The activity of sweet potato PAP on PA FLA was assayed at different pH (4, 5.5, 7, and 7.5) and temperature (25°C, 37°C, and 55°C) conditions. The release of interleukin-8 (IL-8) and the activation of nuclear factor kappa- light-chain-enhancer of activated B cells (NF-κB) in A549 cells exposed to PA FLA treated with or without sweet potato PAP was measured using IL-8 and NF-κB ELISA kits, respectively. The activation of toll-like receptor 5 (TLR5) in TLR5-overexpressing HEK-293 cells exposed to PA FLA treated with or without sweet potato PAP was determined by the secreted alkaline phosphatase-based assay.

**Results:**

The dephosphorylation of PA FLA by sweet potato PAP was favorable at pH 4 and 5.5 and highest at 55°C. PA-FLA treated with the enzyme decreased IL-8 release from A549 cells to about 3.5-fold compared to intact PA FLA at 1,000 ng/mL of substrate. Moreover, PA-FLA dephosphorylated by the enzyme repressed the activation of NF-κB in the cells compared to intact PA FLA. The activation of TLR5 by PA-FLA was highest in TLR-overexpressing HEK293 cells at a substrate concentration of 5,000 ng/mL, whereas PA FLA treated with the enzyme strongly repressed the activation of TLR5.

**Conclusion:**

Sweet potato PAP has the potential to be a new alternative agent against the increased antibiotic resistance of *P. aeruginosa* and may be a new conceptual feed additive to control unwanted inflammatory responses caused by bacterial infections in animal husbandry.

## INTRODUCTION

*Pseudomonas aeruginosa* is a gram-negative bacterium that is widely known to account for up to 10% of all human and animal infections and is one of the main causes of numerous diseases such as hemorrhagic pneumonia, mastitis, urinary tract infections, otitis, and endometritis in domestic animals, causing tremendous damage to animal husbandry [1–3]. Not only does *P. aeruginosa* causes respiratory diseases in animals such as chickens and cattle, but it also causes mastitis in Holstein cows, and according to Banerjee et al [4], mastitis is a known cause of the loss of milk production, which makes it a major problem in the industry [4–7]. Infection with *P. aeruginosa* is stimulated by the microbial molecules called pathogen-associated molecular patterns (PAMPs) [8], and the bacterial flagellin is a well-known PAMP, shown to upregulate pro-inflammatory mediators [9,10]. Also, as it is widely known that the activity and properties of certain proteins vary greatly depending on the phosphorylation of corresponding protein [11], dephosphorylating bacterial flagellin might lead to the regulation of inflammation in flagellin-mediated infections. Structurally, *P. aeruginosa* possesses an unusual surface filament-like flagellum made up of post-translationally phosphorylated flagellin protein [12].

Sweet potato purple acid phosphatase (PAP) is a binuclear metal-containing phosphatase, meaning that it has enzymatically active binuclear metal sites (Fe-Mn center) [13]. The structure of sweet potato PAP is very similar to that of enzyme called phosphoprotein phosphatases (PPPs), binuclear metallohydrolases that catalyze the dephosphorylation of threonine and serine remains [14]. While previous studies indicated that the catalytic cycle and mechanism of PPPs depended upon its crystal structure [15,16], Zhang et al [17] reported the structural similarities of the active sites between PPPs and PAPs, suggesting that PAPs might share the catalytic dephosphorylation mechanism with PPPs. Even though sweet potato PAP had shown phosphatase activity toward eight substrates such as ATP, ADP, AMP, NADP, glucose-6-phosphate, sucrose-6-phophate, fructose-6-phosphate, and p-NPP, studies on the dephosphorylation of sweet potato PAP toward relatively large phosphoproteins are limited [18]. The objective of this study was to investigate the dephosphorylation of bacterial flagellin protein by sweet potato PAP, and the effect of sweet potato PAP on the flagellin-mediated inflammatory response in the A549 lung epithelial cell line.

## MATERIALS AND METHODS

### Reagents and cell culture

Purified flagellin from *P. aeruginosa* (PA FLA) encoded by the *fliC* gene [19], and sweet potato PAP were purchased from InvivoGen (Sandiego, CA, USA) and Sigma-Aldrich (St. Louis, MO, USA), respectively. Both were reconstituted in endotoxin-free water (Sigma-Aldrich, USA). P*i*Color Lock phosphate detection reagent was purchased from Novusbio (Centennial, CO, USA) while the Cymax Human IL-8 ELISA kit was obtained from Ab Frontier (Seoul, Korea). The nuclear factor kappa-light-chain-enhancer of activated B cells (NF-κB) p65 (Total) InstantOne ELISA kit was procured from Invitrogen, Thermo-Fisher Scientific (Carlsbad, CA, USA).

The human alveolar carcinoma epithelial cell line (A549 cells) was procured from American Type Culture Collection (ATCC CCL-185) and was maintained at F-12K Medium Ham’s F-12K (Kaighn’s) Medium (Gibco Life technologies; Gaithersburg, MD, USA) with 10% fetal bovine serum (Gibco Life technologies, USA) and 1% penicillin-streptomycin (Gibco Life technologies, USA) at 37°C in a 5% CO_2_ humidified incubator.

HEK-Blue hTLR5 cells (Invitrogen, Thermo-Fisher Scien tific, USA) were cultured at 37°C in Dulbecco modified eagle medium (DMEM; Gibco Life technologies, USA) with 30 μg/mL of blasticidin (Invitrogen, Thermo-Fisher Scientific, USA) and 100 μg/mL of Zeocin (Invitrogen, Thermo-Fisher Scientific, USA).

### Dephosphorylation of flagellin by sweet potato PAP at various pHs and temperatures

The phosphatase assay was performed at 37°C for 4 hours in a 1-mL reaction mixture containing 4 μL of the enzyme (46.4 μg), 1 μg of PA FLA substrate, and 50 mM Na-acetate (pH 4.0 and 5.5) or Bis-Tris (pH 7.0) or Tris-HCl (pH 7.5). The assay was also performed using the same protocol at conditions of pH 5.5 (Na-acetate) for 4 hours at 25°C, 37°C, and 55°C. The released inorganic phosphates (Pi) were read at optical density (OD) 635 nm, using P*i*ColorLock detection kit (Novusbio; Centennial, CO, USA) following the manufacturer’s instructions. Enzyme activity was defined as the amount of enzyme required to liberate 1 nmol of inorganic phosphate per min under the assay conditions divided by mg of the protein.

### Effect of sweet potato PAP on flagellin-induced pro-inflammatory mediators in A549 cells

A549 cells were initially seeded onto 96-well plates at a density of 1.5×10^4^ cells per well and cultured to 80% confluency at 37°C in a 5% CO_2_ incubator. Various concentrations of FLA (0, 0.1, 1, 10, 100, 1,000 ng/mL) were treated with 1 μL of sweet potato PAP (11.6 μg) for 6 h. Then, enzyme-treated FLA and intact FLA at each concentration were applied to the cells for 12 h. The levels of interleukin-8 (IL-8) and NF-κB were assayed at OD 450 nm using Cymax Human IL-8 ELISA kit and NF-κB p65 (Total) InstantOne ELISA Kit, respectively, according to the manufacturer’s instructions.

### Effect of sweet potato PAP on flagellin-induced TLR5 activation in TLR5 overexpressing HEK-293 cells

Human TLR5-overexpressing HEK-293 cells (HEK-Blue hTLR5 cells) were cultured in 96-well plates at an initial concentration of 1.5×10^4^ cells per well until 80% confluency. Different concentrations of PA FLA (0, 0.1, 1, 10, 100, 1,000, 5,000 ng/mL) were treated with 1 μL of sweet potato PAP (11.6 μg) for 6 hours. Then, enzyme treated FLA and intact FLA at each concentration were added to the cells. Activated toll-like receptor 5 (TLR5) was monitored at OD 620 nm using the secreted alkaline phosphatase-based assay according to the manufacturer’s protocols.

### Statistical analysis

Statistical analyses were conducted using one-way analysis of variance (PROC GLM; SAS 9.4, SAS Inst. Inc., Cary, NC, USA) to search for significant differences between the treatments with Duncan’s multiple range test. The probability level used for statistical significance was p<0.05. The results were presented as the means and standard errors from three experiments.

## RESULTS

### Determination of PA FLA-dephosphorylating activity of sweet potato PAP

**Figure 1:**
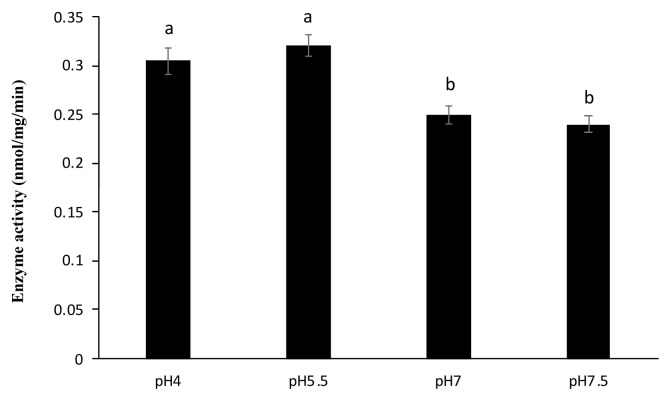
Dephosphorylation of PA FLA by sweet potato PAP in different pH conditions. The phosphatase assay was performed at 37°C for 4 hours in a 1-mL reaction mixture containing 4 μL of the enzyme (46.4 μg), 1 μg of PA FLA substrate, and 50 mM Na-acetate (pH 4.0 and 5.5) or Bis-Tris (pH 7.0) or Tris-HCl (pH 7.5). Enzyme activity was defined as the amount of enzyme required to liberate 1 nmol of inorganic phosphate per min divided by mg of the protein. The data were expressed as the mean and standard errors from three experiments. PA FLA, *Pseudomonas aeruginosa* flagellin; PAP, purple acid phosphatase. ^a,b^ Means lacking common letters differ significantly (p<0.05).

**Figure 2:**
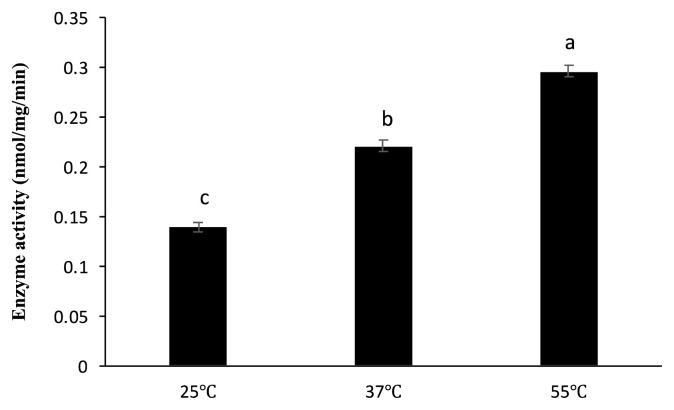
Dephosphorylation of PA FLA by sweet potato PAP in different temperatures. The phosphatase assay was performed in a 1-mL reaction mixture containing 4 μL of the enzyme (46.4 μg) and 1 μg of PA FLA substrate at pH 5.5 (Na-acetate) for 4 hours at 25°C, 37°C, and 55°C. Enzyme activity was defined as the amount of enzyme required to liberate 1 nmol of inorganic phosphate per min divided by mg of the protein. The data were expressed as the mean and standard errors from three experiments. PA FLA, *Pseudomonas aeruginosa* flagellin; PAP, purple acid phosphatase. ^a–c^ Means lacking common letters differ significantly (p<0.05).

As shown in Figure 1, the dephosphorylation of PA FLA by sweet potato PAP was more effective in acidic conditions (pH 4 and 5.5) compared to neutral conditions (pH 7 and 7.5). Moreover, the phosphatase activity was highest at 55°C (Figure 2).

### Effect of sweet potato PAP on PA FLA-induced IL-8 secretion in A549 cells

**Figure 3:**
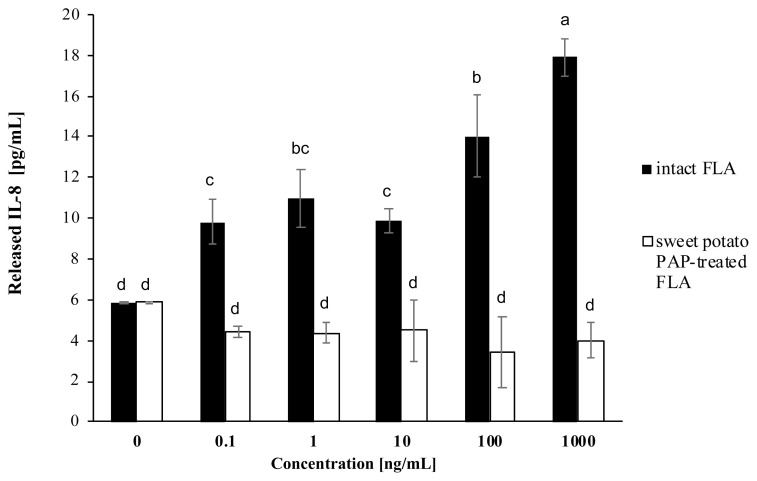
Effect of sweet potato PAP on PA FLA-induced IL-8 secretion in A549 cells. A549 cells were initially seeded onto 96-well plates at a density of 1.5×10^4^ cells per well and cultured to 80% confluency at 37°C in a 5% CO_2_ incubator. Various concentrations of FLA (0, 0.1, 1, 10, 100, 1,000 ng/mL) were treated with 1 μL of sweet potato PAP (11.6 μg) for 6 h. Then, enzyme-treated FLA and intact FLA at each concentration were applied to the cells for 12 h. The level of IL-8 was assayed at OD 450 nm using Cymax Human IL-8 ELISA kit. The data were expressed as the mean and standard errors from three experiments. PAP, purple acid phosphatase; PA FLA, *Pseudomonas aeruginosa* flagellin; IL-8, interleukin-8; OD, optical density; ELISA, enzyme-linked immunosorbent assay. ^a–d^ Means lacking common letters differ significantly (p<0.05).

As shown in Figure 3, PA-FLA stimulated the release of IL-8 in A549 cells. However, PA-FLA treated with sweet potato PAP inhibited the secretion of IL-8 in the cells, decreasing the IL-8 release to about 3.5-fold compared to that of intact PA-FLA, even at 1,000 ng/mL of substrate.

### Effect of sweet potato PAP on PA FLA-induced NF-κB activation in A549 cells

**Figure 4:**
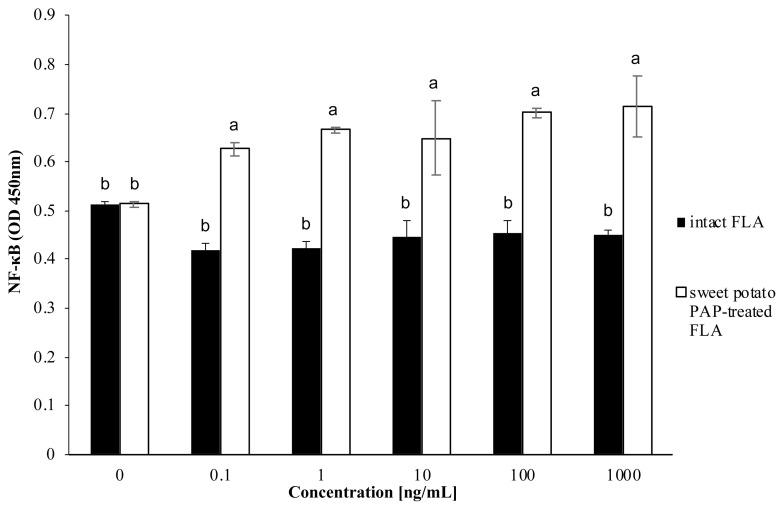
Effect of sweet potato PAP on PA FLA-induced NF-κB activation in A549 cells. A549 cells were initially seeded onto 96-well plates at a density of 1.5×10^4^ cells per well and cultured to 80% confluency at 37°C in a 5% CO_2_ incubator. Various concentrations of FLA (0, 0.1, 1, 10, 100, 1,000 ng/mL) were treated with 1 μL of sweet potato PAP (11.6 μg) for 6 h. Then, enzyme-treated FLA and intact FLA at each concentration were applied to the cells for 12 h. The level of NF-κB was assayed at OD 450 nm using NF-κB p65 (Total) InstantOne ELISA Kit. The data were expressed as the mean and standard errors from three experiments. PAP, purple acid phosphatase; PA FLA, *Pseudomonas aeruginosa* flagellin; NF-κB, nuclear factor kappa- light-chain-enhancer of activated B; OD, optical density; ELISA, enzyme-linked immunosorbent assay. ^a,b^ Means lacking common letters differ significantly (p<0.05).

Figure 4 showed that PA FLA dephosphorylated by sweet potato PAP repressed the activation of NF-κB in A549 cells compared to intact PA FLA, but there was no significant difference between the substrate levels.

### Effect of sweet potato PAP on PA FLA-induced TLR5 activation in TLR5 overexpressing HEK-293 cells

**Figure 5:**
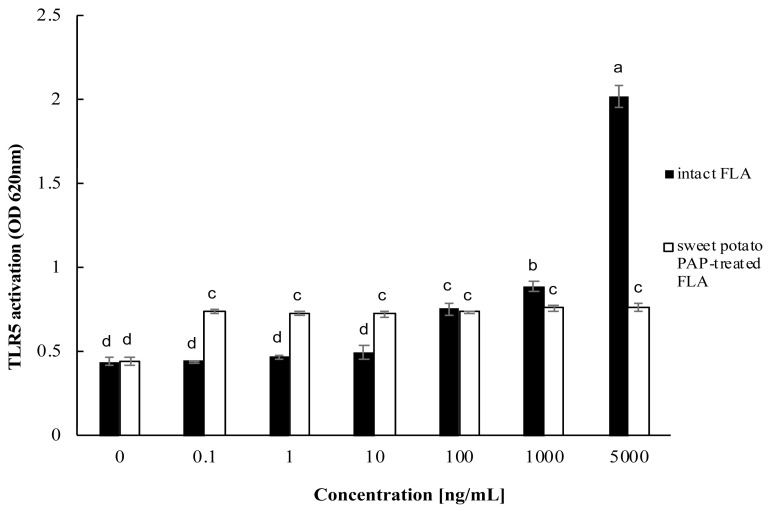
Effect of sweet potato PAP on PA FLA-induced TLR5 activation in HEK-Blue hTLR5 cells. HEK-Blue hTLR5 cells were cultured in 96-well plates at an initial concentration of 1.5×10^4^ cells per well until 80% confluency. Different concentrations of PA FLA (0, 0.1, 1, 10, 100, 1,000, 5,000 ng/mL) were treated with 1 μL of sweet potato PAP (11.6 μg) for 6 hours. Then, enzyme treated FLA and intact FLA at each concentration were added to the cells. Activated TLR5 was monitored at OD 620 nm using the SEAP-based assay. The data were expressed as the mean and standard errors from three experiments. PAP, purple acid phosphatase; PA FLA, *Pseudomonas aeruginosa* flagellin; TLR5, toll-like receptor 5; HEK-Blue hTLR5 cells, human TLR5-overexpressing HEK-293 cells; OD, optical density; SEAP, secreted alkaline phosphatase. ^a–d^ Means lacking common letters differ significantly (p<0.05).

As shown in Figure 5, the activation of TLR5 by PA FLA was effective in TLR5-overexpressing HEK293 cells at substrate concentrations over 100 ng/mL and was the highest at a substrate concentration of 5,000 ng/mL, where PA FLA treated with sweet potato PAP strongly repressed the activation of TLR5.

## DISCUSSION

*P. aeruginosa* infections cause animal diseases and pose a great threat to animal husbandry. The bacteria is responsible for various respiratory diseases, mastitis, otitis, and many others [1,3]. However, the frequently used antibiotics have allowed these bacteria to acquire antibiotic resistance [20,21]. Thus, the importance of finding new alternatives targeting antibiotic resistant bacteria like *P. aeruginosa* has grown. Although flagellin had been reported to be a PAMP in *P. aeruginosa*, there are insufficient studies regarding the inactivation of PA FLA.

Several studies on the functions of mammalian PAP have already been conducted and suggested that PAP was involved in physiological events like osteoclastic bone resorption and erythrophagocytosis through the dephosphorylation of certain proteins [22]. Nevertheless, a precise study on the dephosphorylating activity of sweet potato PAP with relatively high molecular weight substances like proteins has not yet been reported. In the present study, sweet potato PAP successfully exhibited phosphatase activity against PA FLA (Figures 1, 2), which was a novel finding. Moreover, sweet potato PAP was highly active at acidic pH range (4 to 5.5) (Figure 1), which was compatible with the result of chickpea PAP (CaPAP7), verifying the typical property of acid phosphatase [23]. Previously, the phosphatase activity of sweet potato PAP for the substrate, *p*-nitrophenyl phosphate (pNPP) exhibited the highest activity at 50°C [18], which is almost similar to our result of 55°C for PA PLA (Figure 2).

Indeed, bacterial flagellins elicit pro-inflammatory cyto kines such as IL-6 and IL-8 in various cell types [24–27]. As shown in Figure 3, PA FLA provoked the secretion of the universal inflammatory marker IL-8 in A549 cells. However, treatment of the cells with every concentration of substrate dephosphorylated by sweet potato PAP reduced the secretion of IL-8, suggesting that the enzyme exerted anti-inflammatory activity. To some extent, the dephosphorylation of flagellin appears to be related to dysfunction. For example, dephosphorylation of the profission Drp1 (dynamin-related protein 1) protein by the cytosolic phosphatase calcineurin led to the fragmentation of depolarized mitochondria during the cell cycle, differentiation, and death [28].

Moreover, PA FLA dephosphorylated by the enzyme re pressed the activation of NF-κB (Figure 4), which is widely known to mediate *IL-8* gene expression [29]. Bacterial flagellin provokes inflammation via stimulating the NF-κB pathway [30,31].

TLRs regulate the innate immune response and contribute to enhancing antibacterial defenses in host cells [32]. Among the TLRs, TLR5 is well-known to recognize FLA on the bacterial cell surface, eliciting inflammatory signaling by activating the NF-κB pathway [31]. When a bacterial infection takes place, host cells over-produce TLR5 to defend against such attacks [32].

As shown in Figure 5, intact PA FLA clearly induced the activation of TLR5 in TLR5- overexpressing HEK-293 cells at higher doses of more than 1,000 ng/mL, which was in good agreement with the previous result observed in 293T cells exposed to purified *Salmonella typhimurium* flagellin [33], but PA FLA dephosphorylated by sweet potato PAP repressed it. Bacterial flagellins structurally possess two highly conserved N-terminal and C-terminal domains and one central hypervariable domain [32]. In the case of *Salmonella enterica*-derived flagellin, one variant with a deletion in the N-terminus (FliCΔ1–180) failed to stimulate TLR5 [32]. Presumably, phosphotyrosine exists within the N-terminus of PA FLA and the dephosphorylation of the residue inhibited the interaction of PA FLA with TLR5 [12].

Just as exogenous bovine intestinal alkaline phosphatase has been extensively applied to clinical human trials [34], the conception of the administration of sweet potato PAP by oral delivery or by intravenous injection can be positively suggested to maintain good health for farm and companion animals against the gastrointestinal inflammatory disorder and antibiotics-related infections [34,35]. In conclusion, sweet potato PAP has the potential to be a new alternative agent against the increased antibiotic resistance of P*. aeruginosa* and may be a new conceptual feed additive to control unwanted inflammatory responses caused by bacterial infections in animal husbandry.
